# Impact of dietary salicylates on angiogenic factors and biochemical parameters in a rat model of preeclampsia

**DOI:** 10.1371/journal.pone.0333543

**Published:** 2025-09-29

**Authors:** Joanna Suliburska, Rafsan Syabani Cholik, Marta Karaźniewicz-Łada, Dorota Wronka, Anna Karlik, Agnieszka Waśkiewicz, Katarzyna Skrypnik, Paweł Kołodziejski, Adam Cieślak, Łukasz Przybył

**Affiliations:** 1 Department of Human Nutrition and Dietetics, Poznań University of Life Sciences, Poznań, Poland; 2 Department of Physical Pharmacy and Pharmacokinetics, Poznań University of Medical Sciences, Poznań, Poland; 3 Institute of Bioorganic Chemistry, Polish Academy of Sciences, Poznań, Poland; 4 Department of Chemistry, Poznań University of Life Sciences, Poznań, Poland; 5 Department of Animal Physiology, Biochemistry and Biostructure, Poznań University of Life Sciences, Poznań, Poland; 6 Department of Animal Nutrition, Poznań University of Life Sciences, Poznań, Poland; University of Calabria, ITALY

## Abstract

**Background:**

The pathophysiology of preeclampsia involves impaired cytotrophoblastic invasion, placental ischemia, inflammation, and angiogenic imbalance. Prophylactic low-dose aspirin can reduce the risk of preeclampsia and fetal growth restriction in high-risk women. This study evaluated the effect of dietary salicylates on the development of preeclampsia in rats treated with L-NAME (NG-nitro-L-arginine-methyl ester).

**Methodology:**

Pregnant Sprague-Dawley rats were randomly assigned to six groups and treated with dietary salicylates at two dose levels (1 and 10 mg/kg diet) or aspirin (doses adjusted to dietary salicylates). Preeclampsia was induced by administering L-NAME in drinking water from gestational days 6–19.

**Results:**

Neither dietary salicylates nor aspirin, at either dose, affected blood pressure in L-NAME-treated rats. The lower dose of dietary salicylates significantly reduced urinary albumin levels. Both interventions prevented an increase in the sFlt/PLGF ratio and mitigated histopathological placental changes in preeclamptic rats. The higher dose of aspirin reduced placental VEGFR2 protein levels.

**Conclusion:**

Dietary salicylate supplementation does not provide clear preventive effects against preeclampsia.

## Introduction

Preeclampsia is a severe pregnancy complication and a leading cause of maternal and perinatal mortality and morbidity. Newborns of preeclamptic women often experience intrauterine growth restriction (IUGR) and preterm birth, along with elevated fetal proinflammatory profiles that require prolonged and costly hospitalization [1]. The underlying etiology and pathophysiology of preeklampsia remain incompletely understood, but they involve impaired cytotrophoblastic invasion, placental ischemia, and the release of inflammatory and endothelial mediators. Placental dysfunction in preeklampsia is closely associated with angiogenic imbalance [2,3]. Preelampsia is defined by uteroplacental dysfunction, characterized by abnormal angiogenic markers [4]. The main pathophysiological mechanisms include an imbalance between angiogenic and antiangiogenic factors, estrogen deficiency, nitric oxide (NO) synthesis deficiency, increased plasma TNF-alpha, and impaired maternal vascular endothelial function.

Angiogenesis is central to reproductive physiology and the successful progression of pregnancy. During pregnancy, the trophoblast forms the interface between fetal and maternal tissues and serves as a rich source of angiogenic growth factors, including angiopoietins (ANGPT-1, ANGPT-2), placental growth factor (PLGF), and vascular endothelial growth factor (VEGF). Defects in angiogenesis at the maternal–fetal interface contribute to pregnancy disorders, including miscarriage [5]. Recent studies have shown that preeclampsia is associated with altered expression of angiogenic and antiangiogenic factors. In preeclamptic patients, higher levels of soluble fms-like tyrosine kinase 1 (sFlt-1) and lower levels of PLGF and VEGF are observed. Disruption of the VEGF signaling pathway causes endothelial dysfunction across multiple organs, producing hallmark symptoms of preeclampsia such as hypertension, glomerular endotheliosis, and proteinuria [2]. An increased sFlt-1/PLGF ratio and low PLGF alone are considered promising markers for diagnosis and short-term prognosis of preeclampsia in the second trimester [2]. The pathogenesis of preeclampsia is complex. Endothelial dysfunction has been linked to overexpression of storkhead-box protein 1 (STOX1) by the fetoplacental unit [6]. A recent study indicated that STOX1 is a precipitating factor in preterm birth and placental preeclampsia due to early defects in uteroplacental development [7]. Inhibiting STOX1 expression may therefore represent a potential therapeutic strategy.

Randomized clinical trials have shown that the risk of preeclampsia and fetal growth restriction (FGR) can be reduced by prophylactic low-dose aspirin in high-risk women, particularly when administered before 16 weeks of gestation. The exact mechanism by which aspirin prevents preeclampsia remains unclear, but it is hypothesized to reduce deep placentation disorders in women at risk. However, few studies have evaluated the effect of early aspirin use on placentation [8]. Daily low-dose aspirin is known to reduce inflammation by inhibiting cyclooxygenase (COX) activity and decreasing production of the potent vasoconstrictor thromboxane A2 (TxA2). Additionally, it may promote differentiation and inhibit apoptosis in placental cells by improving the cytokine profile [9,10]. Aspirin may also affect angiogenesis in preeclampsia by suppressing angiogenic responses induced by thrombin-activated platelets [11]. Moreover, aspirin has been shown to reduce inflammatory markers, enhance antioxidant capacity, and increase the adhesion of mesenchymal stem cells in the preeclamptic placenta, indirectly benefiting endothelial function and angiogenesis [12]. Its effects may also be strengthened by inhibiting STOX1 expression [13].

Plants synthesize salicylic acid (SA), and salicylates are naturally present in food. Major dietary sources include vegetables, fruits, herbs, and spices [14]. Foods rich in salicylates are thought to contribute significantly to positive health effects [15]. However, it remains unclear whether naturally occurring salicylates provide the same benefits as aspirin. Therefore, this study aimed to evaluate the effect of dietary salicylates on the development of preeclampsia in rats treated with L-NAME.

## Materials and methods

### Animals

Forty-eight female Sprague-Dawley rats (12 weeks old; mean weight: 288 ± 25.52 g) were purchased from Charles River Laboratories, Germany. The study followed the *National Institutes of Health Guide for the Care and Use of Laboratory Animals* (National Institutes of Health Publication No. 80-23, Revised 1978), the European Communities Council Directive of 24 November 1986, and Polish legal requirements. All procedures were approved by the Local Ethics Committee for Animal Experimentation in Poznań (Protocol No. 6/2023). The ARRIVE guidelines (Animal Research: Reporting of In Vivo Experiments) were applied. The rats were acclimated to laboratory conditions for 10 days, with free access to deionized water and a standard AIN-93M diet (Zoolab, Sędziszów, Poland). They were maintained at 21 ± 1°C, 55–65% humidity, and a 12/12-h light/dark cycle, and housed individually in stainless steel cages.

### Experimental design

The experiment was conducted on pregnant rats. Female rats in estrus were housed with male rats for copulation. Pregnancy was confirmed by the presence of a mucus plug in the vagina (gestational day 0, GD0). Pregnant rats were randomly assigned to six groups (*n* = 8 per group):

CH: Healthy control rats
CP: Rats treated with L-NAME (NG-nitro-L-arginine-methyl ester)
LSP: Rats treated with L-NAME and a low dose of dietary salicylates
HSP: Rats treated with L-NAME and a high dose of dietary salicylates
LAP: Rats treated with L-NAME and a low dose of aspirin
HAP: Rats treated with L-NAME and a high dose of aspirin

The explanations of the group designations are as follows: CH refers to the control healthy rats, CP refers to rats with preeclampsia, LSP refers to rats with a low dose of salicylates and preeclampsia, HSP refers to rats with a high dose of salicylates and preeclampsia, LAP refers to rats with a low dose of aspirin and preeclampsia, and HAP refers to rats with a high dose of aspirin and preeclampsia. Preeclampsia induction was performed by providing drinking water containing 0.5 mg/mL L-NAME (N5751, Merck, Sigma-Aldrich, Darmstadt, Germany) from GD6 to GD19. Dietary salicylates and aspirin were incorporated into the feed.

Diets with low and high salicylate content were prepared by adding a mixture of products with relatively high salicylate content to the diets. The composition of the mixture was as follows: buckwheat groats (50%), oregano (10%), basil (10%), cumin (10%), tarragon (10%), and mint leaves (10%). All products were commercially sourced from the Polish market. For the low-salicylate diet (LSP), 1% of the mixture was added to the standard feed; for the high-salicylate diet (HSP), 10% was used. Aspirin doses were matched to the estimated salicylate content of the diets (1 mg/kg for LSP, 10 mg/kg for HSP). The doses of dietary salicylates and aspirin were comparable, as the study aimed to compare the effects of salicylates to those of aspirin (Aspirin®, Bayer) was purchased commercially.

The experimental period spanned GD0 to GD19. Throughout this period, the rats had *ad libitum* access to deionized water and a diet formulated for pregnancy (AIN-93G, Zoolab, Sędziszów, Poland). Food intake was recorded daily, and body weight was measured weekly before food distribution. The full diet composition and salicylate content are presented in Table 1.

**Table 1:** Diets composition.

Group/ Component	CH&CP	LSP	HSP	LAP	HAP
Ash (g/kg dm)	36.56 ± 0.44^a^	37.47 ± 0.49^a^	39.86 ± 0.71^b^	37.41 ± 0.33^a^	37.74 ± 0.43^a^
Protein (g/kg dm)	200.41 ± 0.52^b^	189.01 ± 0.87^a^	185.70 ± 2.01^a^	200.00 ± 3.26^b^	195.38 ± 1.08^b^
Fat (g/kg dm)	75.08 ± 0.11	77.81 ± 0.48	75.91 ± 0.76	76.73 ± 0.16	75.20 ± 1.90
Fiber (g/kg dm)	49.76 ± 1.14^a^	52.74 ± 0.21^b^	53.94 ± 0.54^b^	49.10 ± 1.03^a^	50.08 ± 0.49^a^
NFC (g/kg dm)	642.85 ± 2.75^a^	650.52 ± 1.25^b^	653.09 ± 3.24^b^	643.03 ± 0.51^a^	647.41 ± 0.11^a,b^
Total salicylates (ug/kg dm)	48.79 ± 2.53^a^	1023.14 ± 63.48^b^	11594.97 ± 272.77^c^	1159.94 ± 8.66^b^	12687.22 ± 537.81^c^

dm, dry mass; NFC, non fiber carbohydrates,

a,b,c Significantly differences (*p* < 0.05),

CH, control group, CP, preeclamptic group, LSP, preeclamptic group with low dose of dietary salicylates, HSP, preeclamptic group with high dose of dietary salicylates, LAP, preeclamptic group with low dose of aspirin, HAP, preeclamptic group with high dose of aspirin

At GD19, the rats were weighed and euthanized by decapitation. The decapitation was performed using a rodent guillotine to ensure compliance with animal ethical guidelines and minimize animal suffering to the greatest extent possible.

### Blood pressure measurements

Blood pressure was measured on gestational days 6 (GD6) and 18 (GD18) using a noninvasive blood pressure monitoring system (CODA-PCS42, Kent Scientific, USA). Measurements were taken with a tail cuff equipped with Volume Pressure Recording (VPR) sensor technology. To improve tail blood circulation, the rats were warmed on a heating blanket set at 38°C for 15 min. For each animal, seven readings were recorded and averaged. Before data collection, the rats were habituated to the device and remained calm throughout the inflation–deflation cycles.

### Sample collection

Urine samples were collected on GD6 and GD18 at a consistent morning time, while rats had access to food. Samples were stored at −80°C. Blood was collected in serum separation tubes, allowed to clot at room temperature for 30 min, and centrifuged at 2,000 rpm for 15 min at 4°C. Serum was separated and stored at −80°C. Placentas and fetuses were dissected, weighed, and stored at −80°C. Two placentas per dam were randomly selected and preserved in 10% formalin (Merck, Sigma-Aldrich, Darmstadt, Germany). From three randomly selected fetuses per female, the heart, brain, and liver were dissected and weighed.

### Spleen collection for flow cytometry

Whole spleens were harvested and immediately placed on ice in phosphate-buffered saline (PBS) supplemented with 0.5% bovine serum albumin (BSA) (Sigma-Aldrich) and 2 mM EDTA (Thermo Fisher Scientific). Splenocytes were freshly isolated by mechanically dissociating the tissue through a 70 μm cell strainer. Red blood cells were lysed using eBioscience RBC Lysis Buffer (Thermo Fisher Scientific) for 4 min at room temperature. The remaining cells were washed with 20 mL PBS containing 0.5% BSA and 2 mM EDTA and filtered through a 40 μm cell strainer. Isolated cells were resuspended in 3 mL fetal bovine serum (FBS) (EurX) containing 10% DMSO (Sigma-Aldrich), transferred to cryovials, and frozen at −80°C in a CoolCell freezing container (Corning) for 24 h. For long-term storage, cryovials were transferred to the vapor phase of a liquid nitrogen tank.

### Staining and flow cytometry

Frozen splenocytes were thawed in a 37°C water bath for 2 min and immediately transferred into 5 mL of prewarmed Dulbecco’s Modified Eagle Medium (DMEM) (Lonza). Cells were centrifuged at 300 g (~1300 rpm) for 5 min, resuspended in 4 mL DMEM, and counted using a TC20 automated cell counter (Bio-Rad). One million viable splenocytes were placed on ice, washed twice with PBS, and stained with Ghost Dye V510 viability dye (Tonbo Biosciences) at a 1:1000 dilution in PBS for 30 min on ice. Cell viability was assessed both before freezing and after thawing using Trypan blue exclusion and the TC20 automated counter. Following viability staining, cells were washed twice with PBS supplemented with 0.5% BSA and 2 mM EDTA. For Fc receptor blocking, cells were incubated with anti-CD32 antibody (1:100, BD Biosciences, cat. 550270) and then stained on ice for 30 min with antibody cocktails prepared in PBS containing 0.5% BSA and 2 mM EDTA. The following antibodies were used: CD3 VioBlue (1:50, Miltenyi Biotec, cat. 130-123-874), CD4 Super Bright 600 (1:20, Thermo Fisher Scientific, cat. 63-0040-82), CD8 Super Bright 780 (1:20, Thermo Fisher Scientific, cat. 78-0084-82), CD161 PerCP-eFluor 710 (1:16, Thermo Fisher Scientific, cat. 46-1610-82), CD45R APC-eFluor 780 (1:25, Thermo Fisher Scientific, cat. 47-0460-82), and His48 FITC (1:33, Thermo Fisher Scientific, cat. 11-0570-82). After staining, cells were washed twice with PBS containing 0.5% BSA and 2 mM EDTA and analyzed on a NovoCyte 3000 flow cytometer (Agilent). Data were processed with FlowJo v10 software. Immune cell populations were identified based on surface markers as follows: T lymphocytes: CD3 ⁺ , Helper T lymphocytes: CD3 ⁺ CD4 ⁺ , Cytotoxic T lymphocytes: CD3 ⁺ CD8 ⁺ , Natural killer cells (NK): CD3 ⁻ CD161 ⁺ , B lymphocytes: CD3 ⁻ CD45R ⁺ , Neutrophils: CD3 ⁻ His48 ⁺ SSC^hi^.

### VEGFR2 immunohistochemical staining methodology

Placental tissues were trimmed, processed through graded alcohol and xylene in a tissue processor, and embedded in paraffin blocks. Sections were placed on slides, heated at 56°C for 30 min, and incubated in xylene for 10 min. Slides were then immersed in 99% and 95% ethanol for 10 min each, followed by two 5-min washes in distilled water.

Antigen retrieval was performed with SignalStain® Citrate Unmasking Solution (Cell Signaling, #14746) by heating at 98°C for 10 min, then incubating for 30 min at room temperature in the same buffer. Immunostaining was conducted using the Rabbit Specific HRP/DAB Detection IHC Kit (Abcam, #ab64261). Sections were blocked with hydrogen peroxide for 10 min, washed twice with TBST, and further blocked with 4% skimmed milk in TBST for 10 min, followed by two additional TBST washes.

Slides were incubated overnight at 4°C with primary anti-VEGF Receptor 2 antibody (1:100, Abcam, #ab2349-500) diluted in TBST buffer. Slides were incubated overnight at 4°C, then washed three times with TBST buffer and incubated with a biotinylated secondary antibody, Goat Antipolyvalent, for 10 min, followed by two more washes with TBST buffer. Slides were then incubated with streptavidin peroxidase for 10 min and washed again twice with TBST buffer. Staining was visualized using DAB, prepared 1 min before use. The reaction was stopped once color development was visible (10–30 s, depending on tissue type). Finally, slides were washed in distilled water and counterstained with eosin.

### VEGFR2 measurements

To evaluate VEGFR2 expression, slides were scanned using a PANNORAMIC 250 Flash III scanner (3DHISTECH, Hungary). The mean chromogen concentration (DAB) and mean optical density were quantified with QuPath software. Measurements were performed separately for five anatomical regions of each placenta: decidua, junctional zone, labyrinth, chorionic plate, and yolk sac (if present). For each region, the largest visibly identifiable area was analyzed, with minimum areas defined as follows: yolk sac (42,320 μm^2^), labyrinth (6,902,476 μm^2^), junctional zone (1,555,031 μm^2^), decidua (429,099 μm^2^), and chorionic plate (480,609 μm^2^). Within each region, both mean chromogen concentration and mean optical density were recorded [16].

### Measurements of the diameter of the vessels in the villi

Histological slides stained with hematoxylin and eosin were scanned using a PANNORAMIC 250 Flash III scanner (3DHISTECH, Hungary). To assess vessel lumen diameter in villi, 8–10 blood vessels were selected from the villous region of the placenta. Using QuPath, the vessel diameter was measured at the widest cross-section by drawing a line across the lumen and recording its length.

### Measurements of the light surface of the labyrinth

To assess the vascular lumen area within the labyrinth, Trichrome-Masson staining was performed to provide a strong contrast between labyrinth tissue and vessel lumen. Stained slides were scanned with a PANNORAMIC 250 Flash III scanner, and lumen areas were measured using the QuPath program. Within labyrinth tissue, eight compact squares (~1000 μm^2^ each) without empty spaces were selected. The following QuPath function sequence was applied: *Classify > Pixel Classification > Create Threshold* with parameters set as follows: (1) Resolution: Full, (2) Channel: Average Channels, (3) Prefilter: Gaussian, (4) Smoothing Sigma: 2, (5) Threshold: 180 or 225 (adjusted based on tissue observation), and (6) Region: Any annotation ROI. Before classifier application, blue and red intensity values were determined for each tissue to optimize accuracy. After analysis, the ratio of detected tissue to the marked square area was calculated.

### Diet composition

Diet samples were analyzed in triplicate according to AOAC (Association of Official Analytical Chemists) methods for dry matter (DM; method no. 934.01), ash (method no. 942.05), protein (Kjel-Foss Automatic 16,210 analyzer, Foss Electric, Hillerød, Denmark), fat (ether extract, EE; Soxhlet System HT analyzer, Foss Electric, Hillerød, Denmark; method no. 973.18), carbohydrate, and fiber (with amylase and sodium sulfite, expressed without residual ash) using the Fibertech 1020 Analyzer (Foss Analytical AB, Höganäs, Sweden) [17].

### Free and bound salicylic acid determinations in diets

SA was extracted from homogenized diet samples (0.5 g) by vortexing and sonication for 20 min in methanol. After centrifugation, the methanolic extracts were combined, mixed, and split into two equal portions to quantify free SA content and its glucoside (SAG). The glucoside fraction was calculated as the difference between total salicylic acid (TSA) and free SA (TSA–SA). The solvent was evaporated under nitrogen, and the residue was dissolved in 5% trichloroacetic acid. After centrifugation, SA was extracted three times into an organic phase consisting of ethyl acetate, cyclopentane, and isopropanol (100:99:1, v/v/v). For TSA determination, SAG was enzymatically hydrolyzed using 40 units of β-glucosidase in acetate buffer (0.1 M, pH 5.2). The enzyme solution was added to the second portion of the dry residue and incubated for 90 min at 37°C. The reaction was terminated with 5% trichloroacetic acid, and TSA was extracted as described above.

Following solvent evaporation, the residue was dissolved in mobile phase (0.2 M acetate buffer with 0.5 mM EDTA, pH 5.0), filtered through a 0.20 µm syringe filter (Chromafil, Macherey-Nagel, Düren, Germany), and analyzed using an LC chromatograph (Waters, Manchester, MA, USA) equipped with a 2475 Multi-λ Fluorescence Detector (excitation 295 nm, emission 405 nm) (Waters, Manchester, MA, USA) and a Waters HPLC Symmetry C18 column (150 × 3.9 mm, 5 µm particle size) (Waters, Manchester, MA, USA). The mobile phase flow rate was 0.8 mL/min, with a retention time of 8.2 min.

The term *total salicylates* in the diet refers to the sum of all analyzed forms of salicylic acid.

### Determination of total salicylates in rat serum and urine

Concentrations of SA, gentisic acid (GA), and salicyluric acid (SCA) were measured using a validated UHPLC-MS/MS method.

For serum analysis, 200 μL of serum was mixed with 20 µL of 2% formic acid (FA) in methanol, 20 µL of internal standard solution (SA-d4, 1 µg/mL), and 20 µL of 12% FA in water. Liquid–liquid extraction was performed with 2 mL of ethyl acetate. The mixture was shaken for 10 min, centrifuged for 10 min at 3,500 × g, and the organic layer was evaporated at 45°C in a vacuum concentrator (Eppendorf, Germany). The residue was reconstituted in 200 µL of 2% FA in water. For urine analysis, 50 µL of urine was mixed with 50 µL of methanol and 50 µL of SA-d4 (1 µg/mL). Samples were vortexed for 60 s and centrifuged for 10 min at 13,000 g. A 100 μL aliquot of the supernatant was diluted with 100 µL of 2% FA in water.

From both preparations, 10 μL was injected onto a Nexera UHPLC system coupled to an LCMS-8030 Triple Quadrupole tandem mass spectrometer (Shimadzu, Japan). Separation was performed on a Luna 3 µm Phenyl-Hexyl 100 Å LC column (150 × 3 mm, 3 µm; Phenomenex, USA) at 25°C. The mobile phase consisted of water (A) and acetonitrile (B), both containing 0.1% FA. The gradient program was as follows: 0–5 min, linear increase from 20% to 80% B; 5–6 min, 80% B; 6–7 min, decrease from 80% to 20% B; followed by 1 min at 20% B for column equilibration. Flow rate was 0.4 mL/min. MS detection used electrospray ionization, with positive ion mode for GA and negative ion mode for SA, SA-d4, and SCA. Parameters were: desolvation line temperature, 235°C; heat block temperature, 400°C; nebulizing gas flow, 2 L/min; drying gas flow, 10 L/min; and electrospray voltage, 4.5 kV. Analytes were quantified in multiple reaction monitoring (MRM) mode with the following transitions: m/z 137 to 93 (SA), m/z 141 to 97 (SA-d4), m/z 153 to 108 (GA), and m/z 196 to 121 (SCA).

The term *total salicylates* in serum and urine refers to the combined content of all analyzed forms of salicylic acid and its metabolites.

### Biochemical parameters

Serum concentrations of albumin, VEGF, soluble Fms-like tyrosine kinase-1 (sFLT1), PLGF, and tumor necrosis factor alpha (TNF-α) were measured using commercial ELISA kits (Qayee Bio-Technology Co., Ltd., Shanghai, China). Absorbance was read on an Infinite F50 spectrometer (Tecan Group Ltd., Männedorf, Switzerland).

Measurement accuracy was verified by analyzing two standards and comparing calculated values with nominal concentrations. Moreover, three randomly selected rat serum samples were assayed in triplicate, and their concentrations were compared. In all cases, the coefficient of variation was < 5%. Reproducibility was confirmed using a control serum sample provided by the manufacturer.

Placental homogenates were prepared using an automatic homogenizer (MagNA Lyser, Roche, Basel, Switzerland). VEGF, sFLT1, and PLGF levels were quantified in homogenates using the same ELISA kits (Qayee Bio-Technology Co., Ltd., Shanghai, China) and measured by absorbance spectrophotometry (Tecan Group Ltd., Männedorf, Switzerland).

### Western blot analysis

Total proteins were isolated from placental tissue with RIPA buffer. Tissue was homogenized using a Tissuelyzer II (Qiagen, USA), and lysates were centrifuged at 14,000 × g for 15 min at 4°C. Protein concentration was determined using a BCA protein assay kit (Thermo Scientific, Waltham, MA, USA). For electrophoresis, 30 µg of protein was loaded onto a 12% Tris-HCl SDS-PAGE gel, separated, and transferred to a polyvinylidene difluoride (PVDF) membrane. Membranes were incubated overnight at 4°C with primary antibodies against STOX1 (cat. no. CSB-PA765089LA01HU; dilution 1:1000), followed by three washes with Tris-buffered saline. Secondary antibodies were applied for 1 h at room temperature. Protein signals were visualized using the ChemiDoc system (Bio-Rad, USA). Results were expressed as relative protein levels normalized to β-actin.

### Statistical analysis

Statistical analyses were performed using Statistica software, version 13.3 (StatSoft, Tulsa, USA). Data were expressed as mean ± standard deviation (SD) and as median with interquartile range (Q1–Q3). The Shapiro–Wilk test was applied to assess the normality of distribution. For normally distributed variables, group comparisons were performed using one-way ANOVA followed by Tukey’s posthoc test. For nonnormally distributed variables, the Kruskal–Wallis test was applied, followed by multiple comparisons using rank-sum analysis. Differences were considered statistically significant at *p* < 0.05.

## Results

### Diet composition

The addition of dietary salicylate sources significantly increased the total salicylate content in the diet, with the HSP diet containing ten times more salicylates than the LSP diet (Table 1). Aspirin supplementation in the LAP and HAP diets was equivalent to the total salicylate content of the LSP and HSP diets, respectively. Incorporation of dietary salicylate sources also increased fiber and carbohydrate content while decreasing protein levels. The HSP diet had the highest ash content among all groups. Although statistical analysis confirmed significant differences in component levels (based on three replicates with low SD values), the absolute differences were minor, and the plant mixture did not markedly alter the overall nutritional composition of the diets.

### Basic parameters

Average dietary intake and final body weight of pregnant rats were comparable across all groups (Table 2). Total salicylate intake was lowest in the CH and CP groups and highest in the HSP and HAP groups. Significant differences were observed between the low-dose (LSP and LAP) and high-dose (HSP and HAP) groups.

**Table 2:** Final body mass, dietary, and salicylates intake (mean ± SD).

Group/ Parameter	CH	CP	LSP	HSP	LAP	HAP
Body mass (g)	406.25 ± 40.04	392.38 ± 25.31	379.63 ± 27.44	396.00 ± 31.13	406.63 ± 41.02	404.00 ± 24.01
Dietary intake (g)	26.30 ± 2.99	24.37 ± 2.36	24.77 ± 1.87	25.85 ± 2.18	26.13 ± 2.90	26.18 ± 3.25
Total salicylate intake (µg/kg rat)	2.91 ± 0.15^a^	2.93 ± 0.13^a^	61.42 ± 3.81^b^	696.30 ± 16.38^c^	68.57 ± 0.51^b^	756.24 ± 32.06^c^

SD, standard deviation,

a,b,c Significantly different (*p* < 0.05),

CH, control group, CP, preeclamptic group, LSP, preeclamptic group with low dose of dietary salicylates, HSP, preeclamptic group with high dose of dietary salicylates, LAP, preeclamptic group with low dose of aspirin, HAP, preeclamptic group with high dose of aspirin.

Serum salicylate concentrations were significantly higher in the HSP, LAP, and HAP groups compared with the CH, CP, and LSP groups (Table 3). Urinary salicylate levels were also significantly higher in the HSP, LAP, and HAP groups than in the CH and CP groups.

**Table 3:** Total salicylate concentration in serum and urine (median, Q1–Q3).

Group/ Parameter	CH	CP	LSP	HSP	LAP	HAP
Serum total salicylates (ng/ml)	6.56^a^ 4.16–6.81	4.19^a^ 3.70–4.31	11.29^a^ 7.71–14.07	82.01^b^ 51.83–98.79	206.99^b^ 57.00–257.01	2760.05^b^ 1394.75–3912.02
Urine total salicylates (µg/ml)	0.45^a^ 0.34–0.611	0.49^a^ 0.27–0.57	1.44^a,b^ 1.09–1.61	6.66^b^ 4.53–10.51	14.63^b^ 10.76–17.26	87.75^b^ 69.13–131.93

a,b Significantly different (*p* < 0.05),

CH, control group, CP, preeclamptic group, LSP, preeclamptic group with low dose of dietary salicylates, HSP, preeclamptic group with high dose of dietary salicylates, LAP, preeclamptic group with low dose of aspirin, HAP, preeclamptic group with high dose of aspirin.

### Blood pressure and serum albumin concentration

At baseline (GD6), systolic and diastolic blood pressure, as well as urinary albumin levels, were comparable across all groups (Table 4). By GD18, systolic and diastolic blood pressure significantly increased in L-NAME-treated rats (CP group). Low-dose aspirin reduced both systolic and diastolic blood pressure in preeclamptic rats to levels similar to controls. However, dietary salicylates and high-dose aspirin did not significantly affect blood pressure.

**Table 4:** Blood pressure and albumin concentration in urine.

Group/ Parameter	CH	CP	LSP	HSP	LAP	HAP
SYS_GD6 (mmHg)	116.72 ± 12.68	115.48 ± 9.14	119.97 ± 11.51	120.55 ± 8.84	119.96 ± 13.47	120.74 ± 9.67
SYS_GD18 (mmHg)	113.24 ± 14.57^a^	144.39 ± 11.18^b^	150.02 ± 17.39^b^	155.46 ± 9.49^b^	130.88 ± 16.93^a,b^	142.93 ± 9.58^b^
DIA_GD6 (mmHg)	84.73 ± 10.98	84.51 ± 11.69	84.12 ± 10.62	90.01 ± 8.07	89.89 ± 13.38	88.91 ± 11.04
DIA_GD18 (mmHg)	79.57 ± 12.39^a^	102.74 ± 13.85^b^	102.37 ± 18.34^b^	116.04 ± 14.28^b^	89.68 ± 14.53^a,b^	103.56 ± 12.34^b^
Albumin_GD6 (ng/ml)	80.99 ± 5.92	82.38 ± 11.26	87.50 ± 0.10	77.21 ± 11.06	84.31 ± 18.85	86.48 ± 16.94
Albumin_GD18 (ng/ml)	92.99 ± 9.14^a,b^	106.48 ± 11.37^b^	86.79 ± 7.27^a^	110.91 ± 12.35^b^	99.94 ± 10.40^a,b^	96.22 ± 13.84^a,b^

Sys, systolic blood pressure, DIA, diastolic blood pressure, GD6, sixth gestation day, GD18, eighteen gestation day,

a,b Significantly different (*p* < 0.05),

CH, control group, CP, preeclamptic group, LSP, preeclamptic group with low dose of dietary salicylates, HSP, preeclamptic group with high dose of dietary salicylates, LAP, preeclamptic group with low dose of aspirin, HAP, preeclamptic group with high dose of aspirin.

Treatment with L-NAME in the CP group slightly increased urinary albumin compared with controls (Table 4). Rats treated with low-dose salicylates (LSP group) maintained urinary albumin at baseline levels, which were significantly lower than in the HSP and CP groups.

Treatment with L-NAME in the CP group slightly increased albumin concentration in urine compared.

### Fetus, placenta, and related parameters

L-NAME treatment did not affect placental or fetal weight, the mean number of live fetuses, or the mean number of resorptions (Table 5). Subsequent interventions also did not alter these parameters.

**Table 5:** Fetus and placenta parameters.

Group/ Parameter	CH	CP	LSP	HSP	LAP	HAP
Placenta (g)	0.41 ± 0.05	0.42 ± 0.05	0.39 ± 0.04	0.40 ± 0.07	0.38 ± 0.05	0.35 ± 0.04
The mean number of live fetuses (n)	15.00 ± 3.33	14.25 ± 2.25	13.00 ± 2.98	14.57 ± 5.32	14.38 ± 2.67	13.50 ± 1.93
The mean number of resorption (n)	3.00 ± 2.31	1.67 ± 0.58	2.75 ± 1.26	3.33 ± 0.58	2.50 ± 1.73	2.00 ± 0.71
Fetus weight (g)	1.33 ± 0.16	1.36 ± 0.23	1.15 ± 0.09	1.30 ± 0.28	1.22 ± 0.08	1.19 ± 0.04
Liver (% bw)	7.88 ± 0.40	8.10 ± 0.37	7.31 ± 1.22	7.04 ± 2.13	8.18 ± 0.63	8.27 ± 0.56
Brain (% bw)	7.12 ± 0.63	7.11 ± 0.75	7.46 ± 0.77	6.72 ± 2.13	7.71 ± 0.70	7.68 ± 0.39
Heart (% bw)	0.56 ± 0.05	0.51 ± 0.11	0.57 ± 0.10	0.46 ± 0.14	0.56 ± 0.08	0.58 ± 0.06

bw, body weight; *n*, number; CH, control group; CP, preeclamptic group; LSP, preeclamptic group with low dose of dietary salicylates; HSP, preeclamptic group with high dose of dietary salicylates; LAP, preeclamptic group with low dose of aspirin; HAP, preeclamptic group with high dose of aspirin.

The relative weights of the liver, heart, and brain in the preeclampsia-induced group were comparable to those in the control group.

### VEGF concentration and sFLT/PLGF ratio in serum and placenta

Serum VEGF concentrations were unchanged in L-NAME–treated rats (CP group) compared with controls (CH) (Table 6). Neither dietary salicylates nor aspirin affected serum VEGF levels. In the placenta, VEGF content was slightly higher in the CP group than in the CH group. A significant increase in placental VEGF was observed in the LAP group compared with controls.

**Table 6:** Biochemical parameters in serum and placenta (mean ± SD) and morphometric measurement in placenta (median, Q1–Q3).

Group/ Parameter		CH	CP	LSP	HSP	LAP	HAP
Serum	VEGF (pg/ml)	30.90 ± 2.68	28.45 ± 2.28	30.25 ± 1.91	29.05 ± 1.01	28.22 ± 2.01	28.88 ± 1.82
	sFlt/PLGF	0.43 ± 0.05^a,b^	0.51 ± 0.05^c^	0.39 ± 0.04^a^	0.41 ± 0.06^a,b^	0.40 ± 0.03^a,b^	0.48 ± 0.04^b^
	sFlt (ng/ml)	18.74 ± 0.87	19.28 ± 1.41	17.93 ± 1.15	18.19 ± 2.19	19.18 ± 0.57	19.29 ± 1.12
	PLGF (ng/ml)	44.04 ± 4.87	37.92 ± 3.15	45.78 ± 3.18	44.94 ± 4.62	47.92 ± 2.76	40.78 ± 3.35
	TNFα (ng/ml)	13.24 ± 1.67	14.22 ± 0.55	13.64 ± 1.63	13.63 ± 1.23	14.58 ± 0.56	13.83 ± 1.31
Placenta	VEGF (pg/g)	1.40 ± 0.15^a^	1.54 ± 0.11^a,b^	1.53 ± 0.17^a,b^	1.55 ± 0.07^a,b^	1.64 ± 0.11^b^	1.60 ± 0.08^ab^
	sFlt/PLGF	0.36 ± 0.06	0.40 ± 0.06	0.35 ± 0.08	0.36 ± 0.04	0.40 ± 0.03	0.40 ± 0.05
	sFlt (ng/g)	0.85 ± 0.11	0.88 ± 0.08	0.85 ± 0.09	0.86 ± 0.04	0.92 ± 0.07	0.88 ± 0.04
	PLGF (ng/g)	2.40 ± 0.39	2.20 ± 0.22	2.59 ± 0.86	2.38 ± 0.29	2.38 ± 0.26	2.26 ± 0.26
	Vascular area (%)	38.00^a^ 31.20–45.22	44.21^b^ 40.71–47.03	47.32^b^ 36.71–51.50	47.22^b^ 45.50–52.33	42.61^b^ 38.33–46.60	33.72^a^ 30.60–43.50

VEGF, vascular endothelial cell growth factor, sFlt, soluble Fms-like tyrosine, PLGF, placenta growth factor TNFα, tumor necrosis factor alpha,

a,b,c Significantly different (*p* < 0.05),

CH, control group, CP, preeclamptic group, LSP, preeclamptic group with low dose of dietary salicylates, HSP, preeclamptic group with high dose of dietary salicylates, LAP, preeclamptic group with low dose of aspirin, HAP, preeclamptic group with high dose of aspirin.

In serum, the sFlt/PLGF ratio was significantly elevated in L-NAME-treated rats (CP group) compared with controls (Table 6). This ratio decreased significantly in all intervention groups, though it was higher in the HAP group than in the LSP group. Placental sFlt/PLGF ratios were comparable across all groups. The study also analyzed the concentration of TNF alpha in maternal blood serum, but no significant differences in this parameter were found between the groups.

### Angiogenesis parameters measurement in the placenta

**Fig 1:**
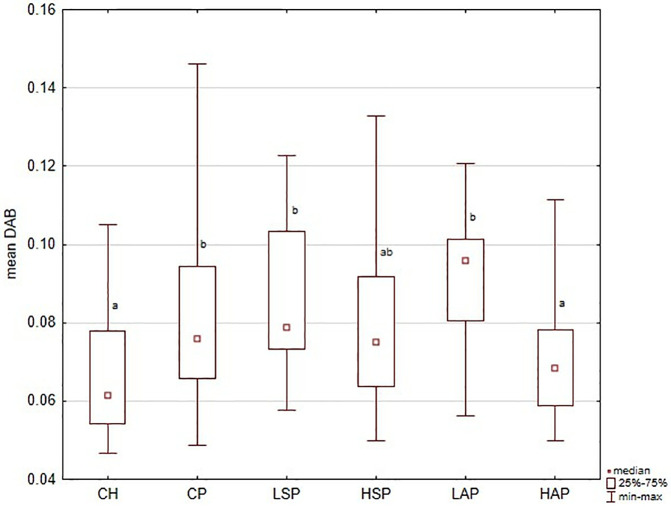
VEGFR2 (vascular endothelial cell growth factor receptor) in the placenta showed as the mean chromogen concentration (DAB); ^a,b^ significantly different between groups (*p* < 0.05).

Analysis of DAB intensity confirmed VEGFR2 expression patterns in the placenta. Results are presented for the whole placenta (Fig 1) and individual regions (). Overall, mean DAB values were significantly higher in L-NAME-treated rats (CP group) compared with controls (CH) (Fig 7). High-dose aspirin (HAP group) reduced DAB values significantly compared with the CP, LAP, and LSP groups.

**Fig 2:**
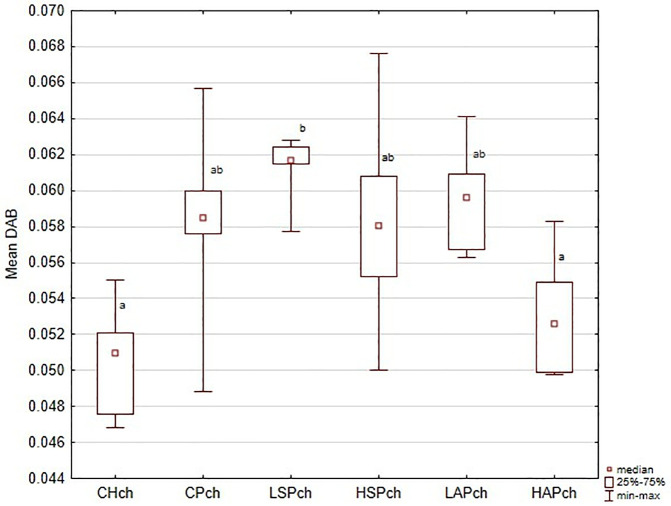
VEGFR2 (vascular endothelial cell growth factor receptor) in chorionic plate localization in placenta showed as the mean chromogen concentration (DAB); ^a,b^ significantly different between groups (*p* < 0.05).

**Fig 3:**
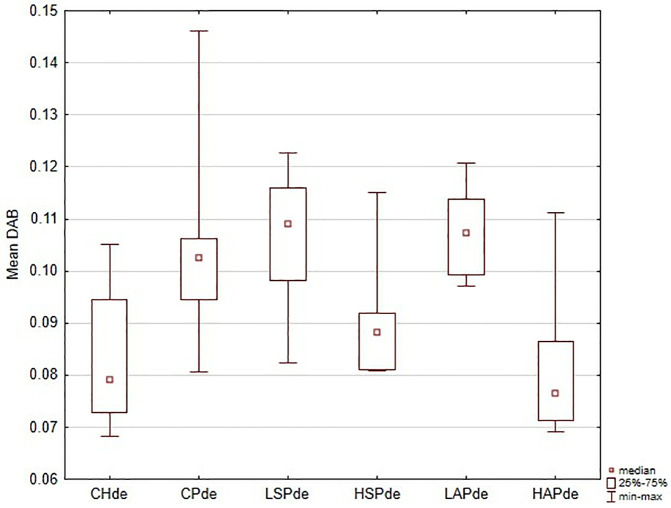
VEGFR2 (vascular endothelial cell growth factor receptor) in decidua localization in placenta showed as the mean chromogen concentration (DAB), lack of significantly differences between groups (*p* > 0.05).

**Fig 4:**
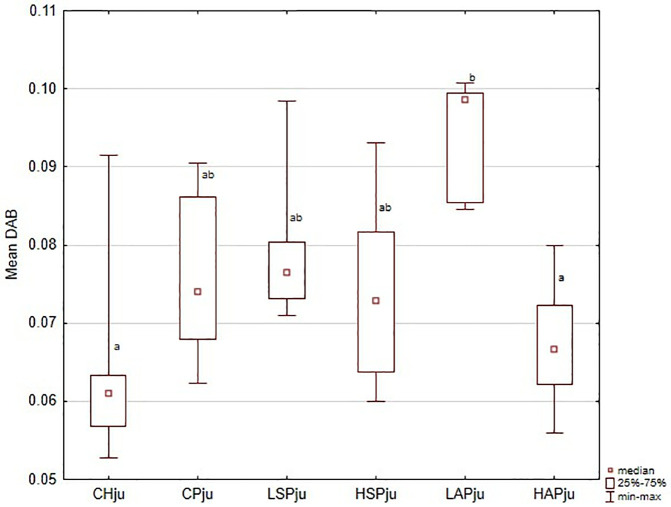
VEGFR2 (vascular endothelial cell growth factor receptor) in junctional zone localization in placenta showed as the mean chromogen concentration (DAB); ^a,b^significantly different between groups (*p* < 0.05).

**Fig 5:**
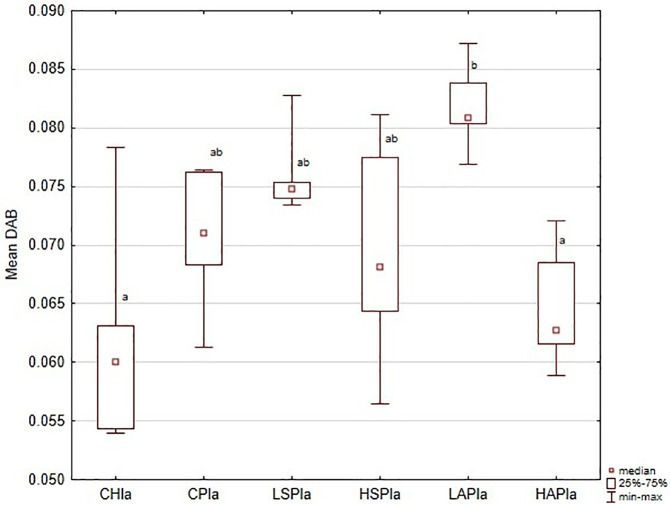
VEGFR2 (vascular endothelial cell growth factor receptor) in labyrinth localization in placenta showed as the mean chromogen concentration (DAB); ^a,b^significantly different between groups (*p* < 0.05).

**Fig 6:**
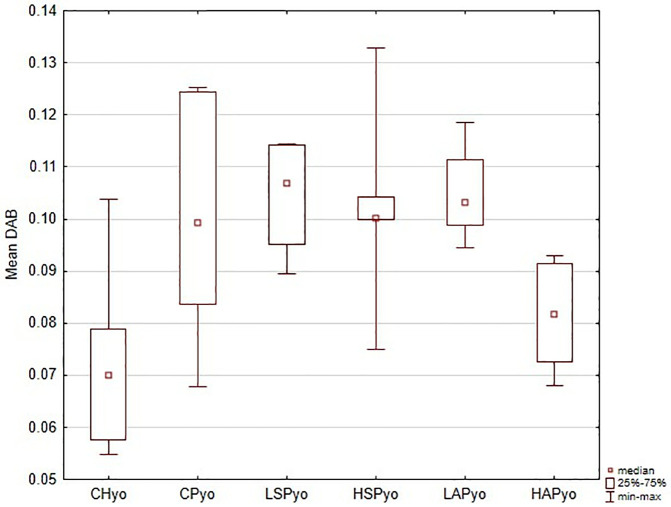
VEGFR2 (vascular endothelial cell growth factor receptor) in yolk sac localization in the placenta showed as the mean chromogen concentration (DAB); lack of significant differences between groups (*p* > 0.05).

**Fig 7:**
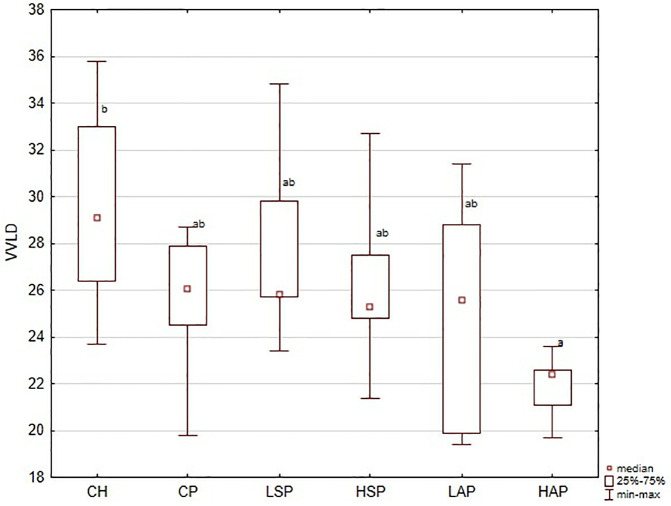
The DAB measurements for VEGFR2 in the placenta in groups (picture above) and villiary vessel luminum diameter (indicated by an arrow) in groups (picture below).

**Fig 8:**
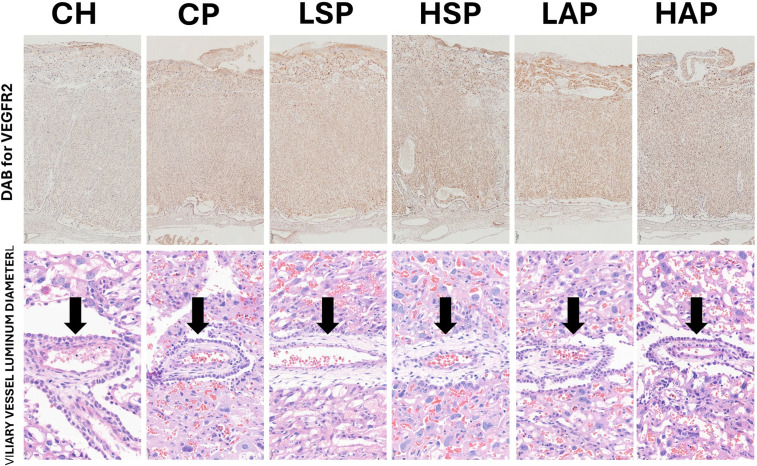
Villiary vessel luminum diameter (VVLD) (µm) in placenta; ^a,b^significantly different between groups (*p* < 0.05).

Regional analysis was consistent with the whole-placenta findings. In general, DAB values were higher in the CP, LSP, HSP, and LAP groups than in the CH and HAP groups (Figs 2–6 and 8). No significant group differences were found in the decidua or yolk sac. In the chorionic plate, DAB intensity was significantly higher in the LSP group than in the CH and HAP groups. In the junctional zone and labyrinth, DAB values were significantly elevated in the LAP group compared with the CH and HAP groups.

**Fig 9:**
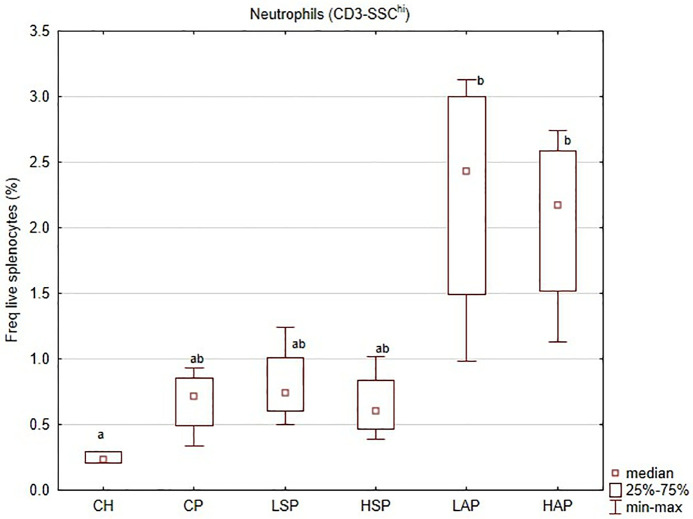
Neutrofil numbers in splenocytes; ^a,b^significantly different between groups (*p* < 0.05).

Villous vessel lumen diameter (VVLD) was also evaluated. VVLD was slightly reduced in the CP group compared with CH (Figs 8 and 9). In the HAP group, VVLD was significantly lower than in the control group.

Placental vascular area results are presented in Table 6. L-NAME treatment significantly increased the vascular area in the CP group compared with CH. Neither natural salicylates nor low-dose aspirin altered this effect. In contrast, high-dose aspirin markedly reduced placental vascular area compared with the CP, LSP, HSP, and LAP groups, as well as the CH group.

### Immunophenotyping of splenocytes

Immunophenotyping was conducted to evaluate whether dietary salicylate intake influences inflammation associated with preeclampsia. Splenocytes collected on GD19 were stained with antibody panels and analyzed by flow cytometry. Comparative analyses included T lymphocytes (and CD4 ⁺ naïve and CD8 ⁺ cytotoxic subsets), B cells, NK cells, and neutrophils (Table 7).

**Table 7:** Spleen immunophenotyping analyses (median, Q1–Q3).

Group/ Parameter		CH	CP	LSP	HSP	LAP	HAP
Freq of live splenocytes (%)	T cells (CD3+)	30.00 27.88–32.10	28.35 27.40–29.10	24.90 24.48–25.70	23.70 23.50–24.25	30.70 29.23–31.98	26.30 24.73–27.70
	CD4 + T cells (CD3 + CD4+)	13.75 13.45–14.78	12.80 11.65–14.58	10.33 9.38–11.18	10.30 10.25–10.35	13.20 11.85–14.38	11.55 10.15–13.00
	CD8 + T cells (CD3 + CD8+)	12.60 12.25–13.70	11.70 10.75–12.80	11.50 11.15–11.60	10.80 10.75–11.25	14.65 14.18–15.13	12.15 11.43–12.85
	B cells (CD3-B220+)	42.90 39.60–47.28	41.70 38.55–45.35	40.10 38.08–41.93	38.45 36.58–39.78	39.90 38.30–40.90	35.40 33.28–37.40
	Natural killer cells (CD3-CD161+)	4.79 3.62–5.77	5.26 4.86–5.79	4.40 4.31–4.55	5.02 4.64–5.42	4.69 4.13–5.03	4.52 4.07–5.29
Freq of live T cells (%)	CD4 + T cells (CD3 + CD4+)	48.75 46.58–50.50	46.20 42.43–51.60	42.80 38.73–44.83	42.60 41.33–43.83	41.80 40.75–43.65	44.85 42.10–46.95
	CD8 + T cells (CD3 + CD8+)	44.25 42.20–46.70	44.15 38.03–48.20	48.25 45.90–52.18	46.50 45.68–43.08	48.40 46.15–50.50	46.55 44.88–48.13

Freq, frequency.

Although L-NAME induced a preeclamptic phenotype based on other parameters, no significant differences were observed between healthy and preeclamptic rats in the levels of T lymphocytes, CD4 ⁺ naïve T cells, CD8 ⁺ cytotoxic T cells, B cells, or NK cells. The only significant change was in neutrophil counts. All L-NAME-treated groups exhibited increased neutrophil numbers, with no additional effect of dietary salicylates. However, aspirin further increased these numbers, nearly tripling the population of neutrophils in the spleen (Fig 9).

### STOX1 protein

**Fig 10:**
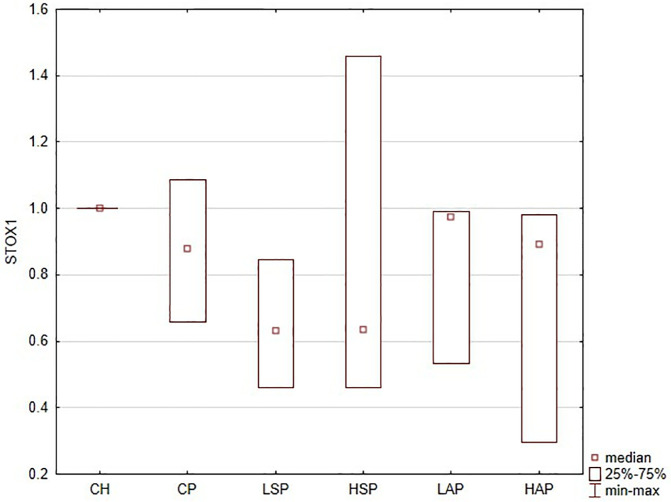
Western blot analysis for STOX1. The results were presented as a relative expression to the reference protein b-actin. A.

Western blot analysis revealed no significant effect of L-NAME treatment or interventions on STOX1 expression in the placenta (Fig 10).

## Discussion

High blood pressure, proteinuria, and an elevated sFlt/PLGF ratio in blood are characteristic indicators of preeclampsia [18]. In this study, these features confirmed successful induction of the preeclampsia model in the CP group compared with the CH group.

This is the first experimental investigation of the effects of dietary salicylates in rats with L-NAME-induced preeclampsia. Our findings demonstrated that salicylate doses obtained from food are insufficient to prevent the development of preeclampsia. From a nutritional perspective, it is difficult to achieve salicylate intakes comparable to those of aspirin used preventively in preeclampsia. Our study confirmed the researchers’ suggestion that the effects of aspirin on various physiological and pathological aspects associated with preeclampsia are dose-dependent. Low-dose aspirin (<100 mg/day in pregnant women) primarily inhibits COX-1, reducing thromboxane A2 synthesis without impairing vascular prostacyclin production [19]. Higher doses appear necessary to influence blood pressure, immune modulation, and angiogenic balance, thereby improving placental vascular function. We initially expected that the effects of natural salicylates in salicylate-rich products would be enhanced by active substances with anti-inflammatory and antioxidant effects [14].

Low-dose aspirin is considered to play a role in the therapy and prevention of preeclampsia. In previous experimental research, a low dose of aspirin is typically defined as 1.5 mg/kg body weight/day, and this dose was often effective in reducing the risk of developing preeclampsia in L-NAME-treated rats [20,21]. This study used much lower doses of aspirin (around 0.07 mg/kg bm and 0.75 mg/kg bm) and did not show significant reductions in preeclampsia parameters, including blood pressure and proteinuria. The relatively low doses of aspirin used in our experiment were related to dietary salicylates, which are present in food at relatively low levels. According to the study design, the aspirin doses matched the salicylate content of the diets. When preparing the diets, it was important to consider both the salicylate content in the food and the order in which the products were added to the diet. This approach was necessary to maintain the composition of the diet while also ensuring the diet remained attractive to the rats, so that dietary intake across the groups did not differ.

We also found no clear correlation between total salicylate intake and its concentrations in blood and urine. Groups receiving dietary salicylates had lower systemic levels than those given aspirin, likely reflecting differences in absorption, metabolism, and dietary matrix effects.

Based on the literature, the average intake of salicylates in a Western mixed diet is 0.1–22 mg/day, and in a vegetarian diet, it reaches 26 mg/day [14]. Therefore, based on the average adult body weight, the daily intake of salicylates ranges from 1 to 400 µg/kg body weight. Low-dose aspirin used prophylactically in women with preeclampsia is < 150 mg/day, which is approximately 2 mg/kg body weight based on body weight. Dietary intake of salicylates in humans is significantly lower, and their bioavailability is limited by other dietary components. It is difficult to determine the specific content/dose of dietary salicylates (especially since the salicylate content in the product usually varies significantly, as it depends on cultivation and processing conditions). Referring to clinical conditions, it is recommended to use a high-salicylate diet, containing products rich in these compounds, and the content in the diet should be checked on an ongoing basis.

Aspirin is rapidly absorbed and has relatively high bioavailability. Although food delays absorption, it does not reduce overall bioavailability [22]. In contrast, salicylates from food are absorbed less efficiently than acetylsalicylic acid. Their uptake may be influenced by interactions with other dietary components and weaker release from the food matrix [23]. Consequently, serum and urinary salicylate concentrations were significantly lower in the LSP and HSP groups than in the LAP and HAP groups, despite comparable salicylate content in the diets. This study showed that dietary salicylates exert much weaker systemic effects than acetylsalicylic acid at equivalent doses.

The L-NAME model, in which preeclampsia is induced by administering L-NAME in drinking water, has been well established in previous studies and consistently reproduces key parameters of preeclampsia [20,21,24]. In this study, L-NAME treatment significantly increased blood pressure, proteinuria, and the sFlt/PLGF ratio, while having no effect on TNFα, STOX1, or FGR. In the placenta, we found a significant increase in VEGFR2 concentration due to L-NAME exposure. VEGFR2 is a parameter that reflects the process of angiogenesis. The increased VEGFR2 content in the placenta was associated with vascular area and a decreased villous vessel lumen diameter. These observed changes suggest abnormal angiogenesis in the placenta. Reduced NO production following L-NAME intake may stimulate compensatory VEGFR2 synthesis, as VEGF strongly promotes placental endothelial NO production [25]. The observed decrease in villous vessel diameter likely reduces placental blood flow. Dysregulation in the placenta leads to the release of angiogenic factors such as sFlt and PLGF into maternal circulation [1,24]. 6 Elevated sFlt levels in preeclampsia have been attributed to VEGF overproduction at the decidual–placental interface, with VEGF shown to stimulate sFlt release from human placental tissue in a dose-dependent manner. In pregnant mice, VEGF treatment induces hypercoagulation and hypertension, mirroring clinical features of preeclampsia [26].

In preeclampsia, excess sFlt reduces the bioavailability of PLGF and VEGF by binding them, thereby decreasing proangiogenic activity. This imbalance contributes to elevated blood pressure and widespread maternal–fetoplacental dysregulation [18,27]. The ratio between sFlt, PLGF, and VEGF is crucial for maintaining a healthy endothelium and normal vascular activity [27,28]. In this model, no significant changes in VEGF concentration were observed. However, the increase in the sFlt/PLGF ratio and VEGFR2 indicates an imbalance in angiogenesis in the placenta. This study found that L-NAME administration caused significant histopathological changes in the placenta, which are associated with improper placental functioning. Increased villi with a thickened basement membrane occur due to ischemia in the uteroplacental circulation, which is linked to placental ischemia [29]. The placenta adapts to hypoxia in variable ways depending on the experimental model, gestational stage, and other factors, leading to differences in angiogenesis and fetal development [30].

Interestingly, treatment with both natural salicylates and aspirin slightly attenuated adverse placental changes. In the LAP and HAP groups, this effect was attributed to SA activity [22,31], while in the LSP and HSP groups, it may have been partly related to the anti-inflammatory and antioxidant properties of active components in the spice mixture [15,23,32]. Aspirin, which is metabolized to SA, can inhibit sFlt expression in trophoblasts by blocking COX-1 and JNK/AP-1 signaling pathways [31]. Additionally, low-dose aspirin inhibits platelet thromboxane-mediated vasoconstriction, thereby protecting against pathological coagulation in the placenta and preventing failure of spiral artery remodeling [33]. Natural salicylates may share the same mechanism of action. Although significant group differences were observed in the serum sFlt/PLGF ratio, absolute concentrations of these factors did not differ significantly. A slight reduction in PLGF was noted in the CP group, while salicylate-treated groups showed a modest increase, approaching control values. This suggests that the reduced sFlt/PLGF ratio observed after salicylate intervention was primarily driven by changes in PLGF concentration, indicating that salicylates may help restore PLGF synthesis impaired under L-NAME conditions.

Both natural salicylates and aspirin significantly reduced the serum sFlt/PLGF ratio, highlighting a potentially beneficial effect. However, serum TNF-α levels remained unchanged, which may reflect minimal systemic anti-inflammatory activity, timing of sampling, or insufficient dosing. Other studies have observed that SA demonstrated beneficial effects on placental blood flow and oxidation, which may indicate a reduction in placental ischemia and an improvement in placental function [15,22,30,31]. In contrast, STOX1 protein levels remained unchanged in all groups, regardless of salicylate treatment.

In this study, no significant effect of dietary salicylates on the main pregnancy outcome and fetal viability parameters was found. Therefore, it appears that the main dietary sources of salicylates, as well as aspirin at the same level in the diet, have no significant impact on the development of preeclampsia.

The results show that the dose of salicylates used (both from food sources and as aspirin) was too low to produce a comprehensive prophylactic effect in L-NAME-treated rats. A slight reduction in blood pressure was observed with low-dose aspirin (LAP group), while low-dose dietary salicylates (LSP group) significantly reduced urinary albumin concentrations, suggesting a possible dose-dependent effect. This effect may also be influenced by other bioactive compounds present in herbs and spices, such as polyphenols and terpenes [14].

When assessing placental angiogenesis, it should be noted that low doses of dietary salicylates and aspirin did not affect VEGFR2. Higher doses of aspirin significantly reduced VEGFR2 in the placenta of L-NAME-treated rats, bringing the mean value for this parameter closer to that of the control group. It is also worth noting that a higher dose of dietary salicylates also decreased VEGFR2, but this change was not statistically significant compared to the CP group. The response to higher doses of salicylates from these different sources may be related to the fact that the rats receiving dietary salicylates had significantly lower concentrations of total salicylates compared to those receiving aspirin. In this study, the response of rats to L-NAME was associated with higher VEGFR2 levels, suggesting that the mechanism of action for the higher dose of aspirin (and dietary salicylates in HSP) may involve a reduction in this proangiogenic parameter, as observed in studies involving increased pathogenic angiogenesis in tissues [31,34–36]. It is also worth noting that the higher dose of aspirin decreased VEGFR2 in all analyzed locations of the placenta, indicating that the effect of aspirin was consistent throughout the placenta. Moreover, the placental vascular area was significantly increased in the CP group and normalized only in the HAP group, suggesting a specific effect of high-dose aspirin.

The results indicate that, under conditions of increased proangiogenic factors, aspirin may inhibit VEGF receptor synthesis, while also reducing the vascular surface area. This effect is opposite to what is expected and may be suggested to be dose-dependent, as relatively low doses of the drug were used in this study [19].

The results of spleen immunophenotyping do not strongly support a role for salicylates in preventing preeclampsia. An increase in splenic neutrophils was observed in all L-NAME groups, consistent with placental ischemia. Unexpectedly, aspirin further increased the number of neutrophils in splenocytes. Previous studies have shown that inflammatory changes vary depending on the preeclampsia model and animal species, and not all models reproduce findings observed in humans [37,38]. In vitro, acetylsalicylic acid has been reported to stimulate the proliferation of human and rat lymphocytes, and low doses of lysine acetylsalicylate increased eosinophil counts in blood and rat splenocytes, likely through enhanced production or recruitment [39]. In women with preeclampsia, leukocytes are activated in circulation, and neutrophils infiltrate maternal vessels. Lipid peroxides, secreted by the placenta, strongly activate leukocytes. Aspirin typically reduces neutrophil thromboxane production and infiltration into maternal vessels [40]. It has been found that NO is a relevant mediator of the aspirin-induced effect on neutrophils and platelet activation. Aspirin affects neutrophils through a NO/cGMP-dependent mechanism. The use of an NO synthase inhibitor in this study may have altered the interaction between aspirin and leukocytes, leading to an increase in neutrophils in splenocytes in the aspirin-treated groups [41].

This study examined the effects of dietary salicylates compared with a drug containing acetylsalicylic acid. Dietary sources of salicylates also contain numerous bioactive compounds with antioxidant, anti-inflammatory, and hepatoprotective properties, as well as effects on angiogenesis [42]. Thus, the outcomes observed in groups receiving spice-enriched diets reflect the combined influence of all plant-derived compounds, not salicylates alone. However, the study did not find a significant effect of dietary salicylates on preeclampsia-related outcomes or fetal viability. Although comparable pharmacological and dietary salicylate levels were tested, no meaningful differences in their effects were observed. It may be assumed that the content and bioavailability of salicylates and other bioactive compounds in consumed spices are too low to significantly affect the analyzed parameters in this rat model of preeclampsia.

This study has several strengths and limitations. A major strength was the wide range of analyses conducted on the collected samples. Moreover, for the first time, the effects of dietary salicylates were investigated in comparison with equivalent doses of aspirin on the development of preeclampsia. A key limitation, however, was that dose matching was based on dietary content rather than systemic exposure. Because serum concentrations differ substantially, this approach limits the comparability between aspirin and salicylate groups. The study would have benefited from dose adjustments to achieve comparable blood levels, enabling a more pharmacologically meaningful comparison. Another limitation was the lack of detailed analysis of the bioactive components present in the salicylate-rich food mixture.

## Conclusion

Dietary salicylates did not influence the development of preeclampsia in L-NAME-treated rats. Because dietary intake of salicylates is low and their absorption from food is limited, they do not exert a clear preventive effect against preeclampsia.
